# Supplementary material for: A Flower-like In2O3 Catalyst Derived via Metal–Organic Frameworks for Photocatalytic Applications
Source: Int J Mol Sci. 2022 Apr 15;23(8):4398. doi: 10.3390/ijms23084398 (PMC9028932; doi:10.3390/ijms23084398)
Supplement: Supplementary file 1 [file ijms-23-04398-s001.zip › ijms-1659617-supplementary.pdf]

International Journal of *Molecular Sciences*

MDPI

Article

A Flower-like In_2O_3 Catalyst Derived via Metal–Organic Frameworks for Photocatalytic Applications

Munisamy Maniyazagan [1], Hyeon-Woo Yang [1], Perumal Naveenkumar [1], Nayoung Kang [1], Woo Seung Kang [2] and Sun-Jae Kim [1,*]

[1] Department of Nanotechnology and Advanced Materials Engineering, Sejong University, Seoul 05006, Korea; manichemist@gmail.com (M.M.); hyunket@naver.com (H.-W.Y.); pcnaveenperumal@gmail.com (P.N.); nykang0615@naver.com (N.K.); wkang651@inhatc.ac.kr (W.S.K.)

[2] Department of Metallurgical and Materials Engineering, Inha Technical College, Incheon 22212, Korea

1. Photocatalytic activity

1.1. Degradation of MB

The photocatalytic activities of prepared samples were evaluated through degradation of Methylene blue. 0.2 g of In_2O_3-MF catalyst was added to 50 mL of MB dye (30 mg L^{-1}) and stirred under UV–Vis light. Before UV–Vis light irradiation, the suspension was magnetically stirred in the darkness for 30 min to keep the adsorption–desorption equilibrium. By monitoring the absorption bands of MB at 664 nm, the relationship between reaction progress and time can be determined.

1.2. Reduction of 4-NP to 4-AP

An aqueous solution of p-nitrophenol (10^{-4} M) and fresh $NaBH_4$ (10^{-3} M) were added into a beaker. 0.3 g of the In_2O_3-MF catalyst was then added to the mixture to begin the reduction reaction. The reaction progress was followed via UV–vis spectroscopy. 4-NP has a maximum absorption peak at 318 nm. Upon the addition of little amount $NaBH_4$, this peak change to 400 nm due to the formation of phenolate ion. After reduction of the phenolate ion, the peak at about 400 nm is flattened and the amine peak appears at 300 nm. In order to evaluate the reaction rate, the concentration chart was plotted against the absorption.

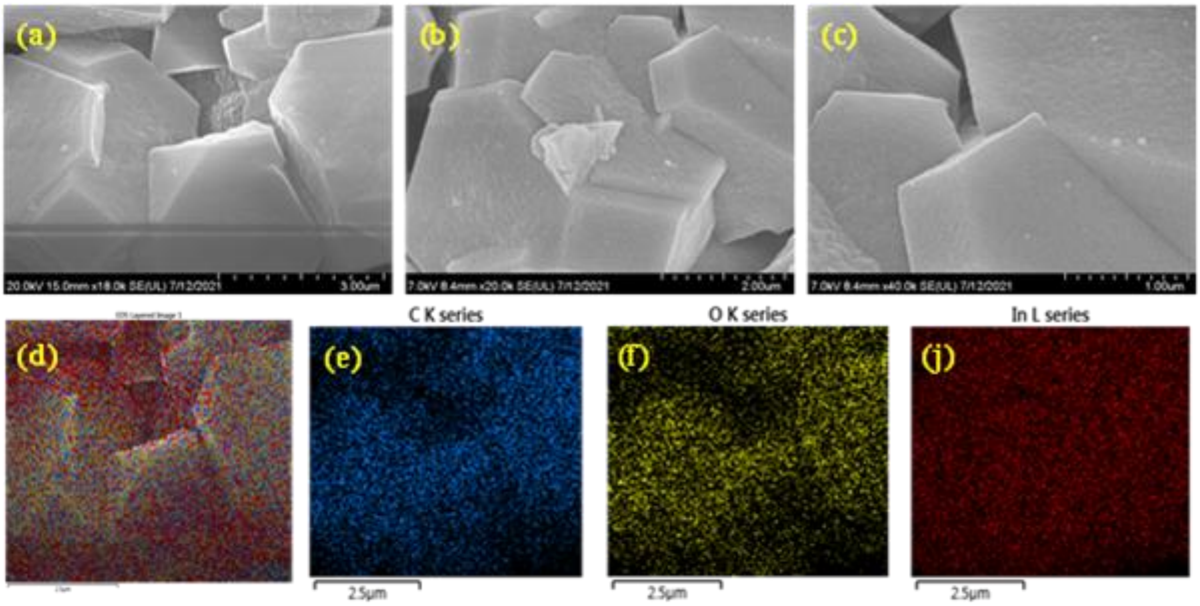

Figure S1. SEM image (a-c), element mappings of In_2O_3-MD (d-j).

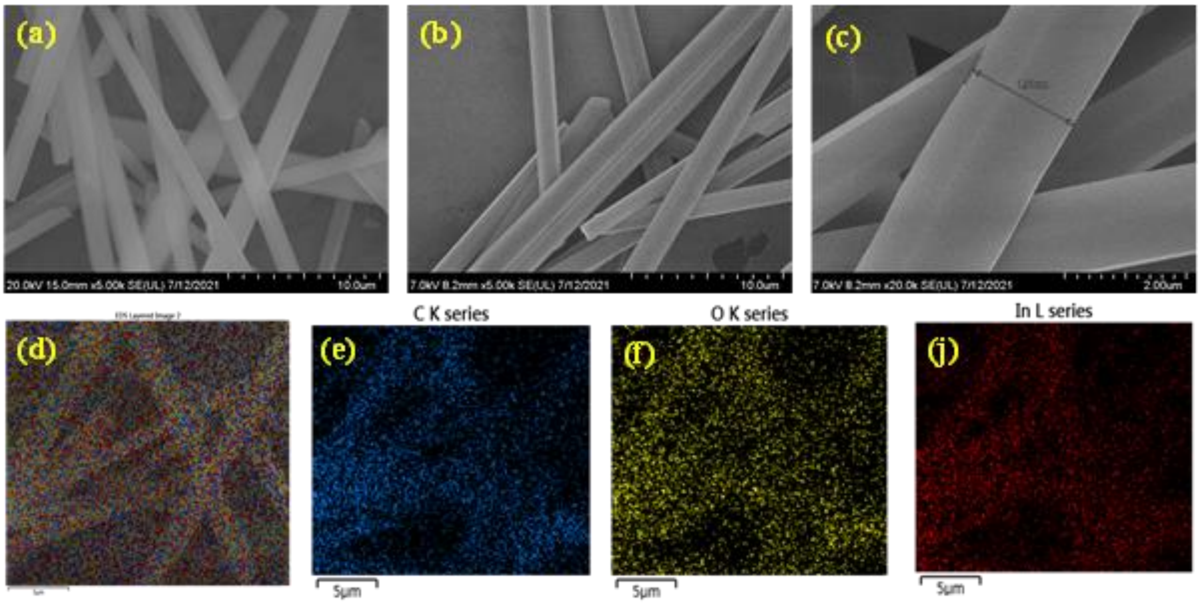

Figure S2. SEM image (a-c), element mappings of In_2O_3-MR (d-j).

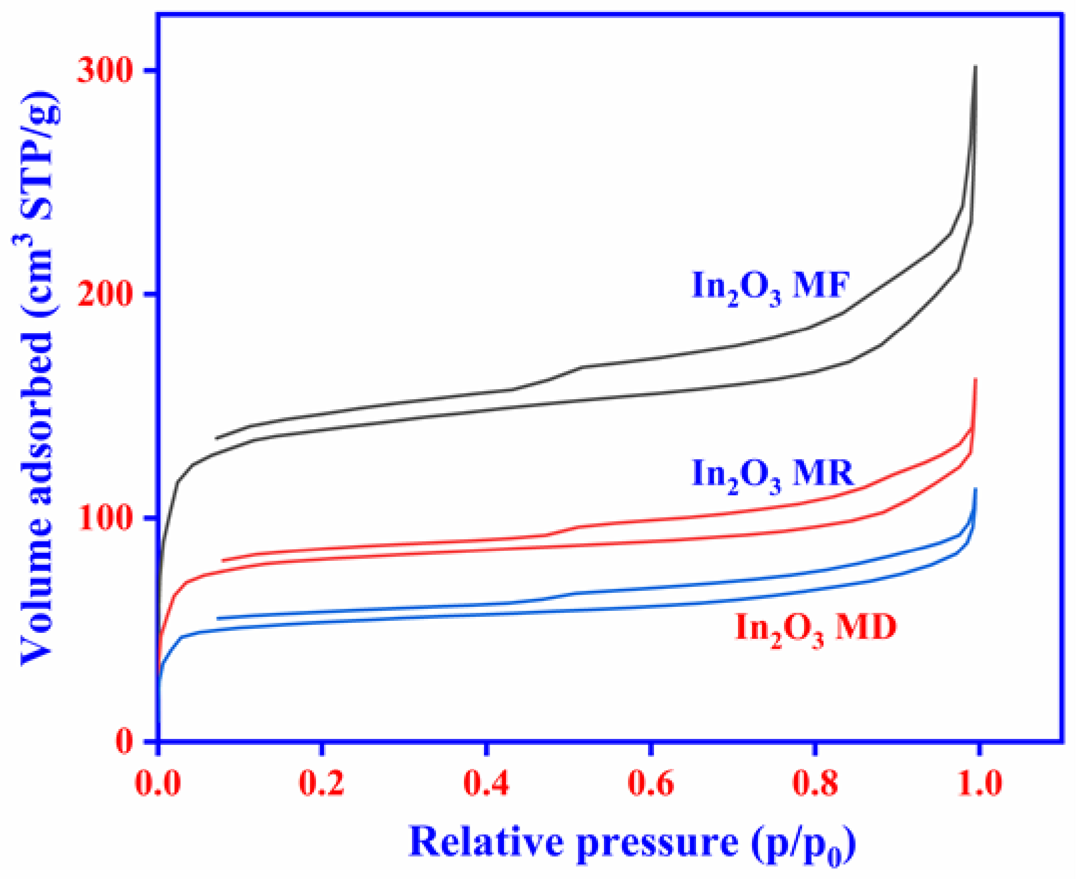

Figure S3. BET nitrogen adsorption–desorption isotherms of In_2O_3-MD, In_2O_3-MR and In_2O_3-MF.

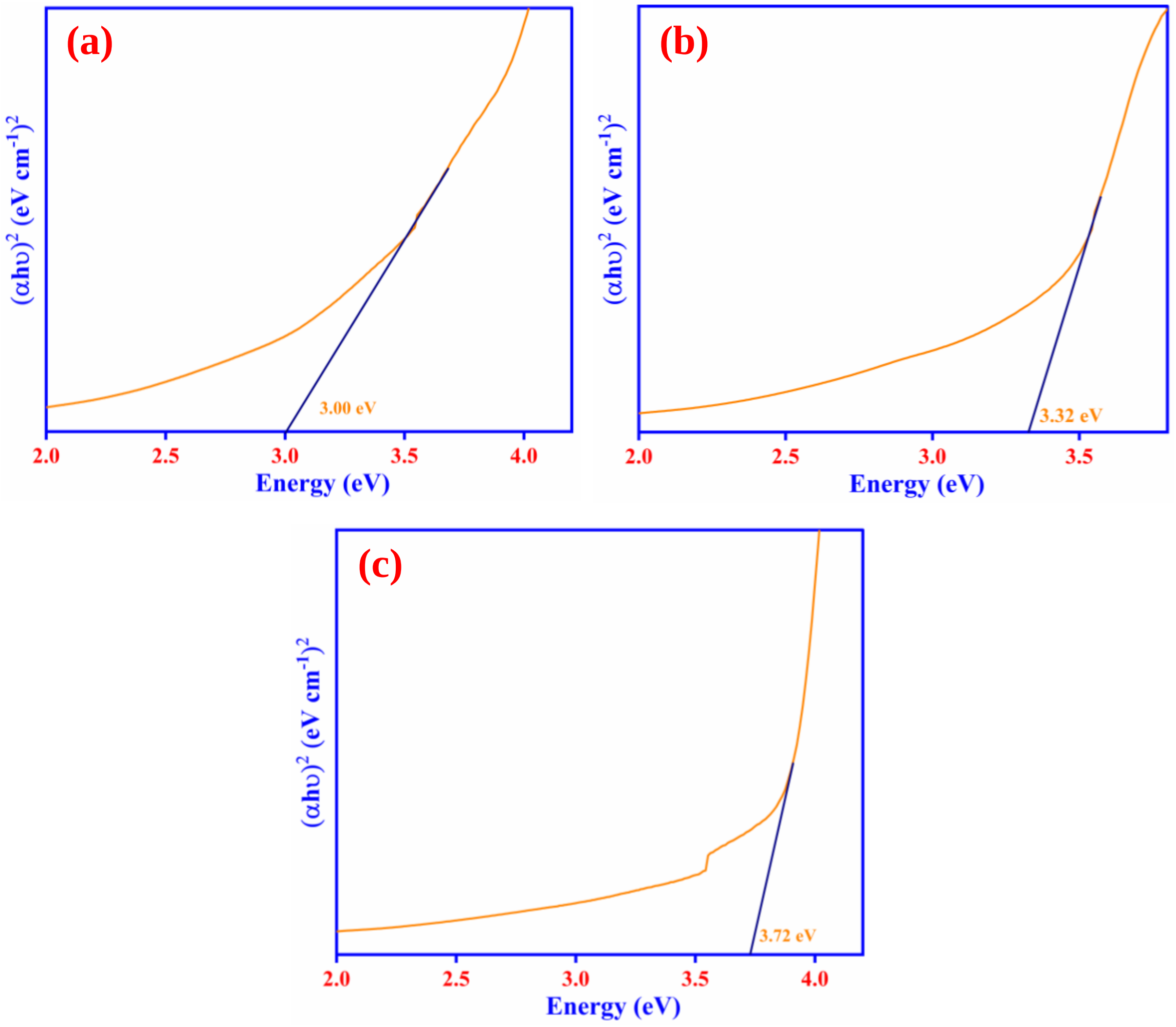

Figure S4. Tauc's plots of the (a) In_2O_3-MF, (b) In_2O_3-MR, (c) In_2O_3-MD.

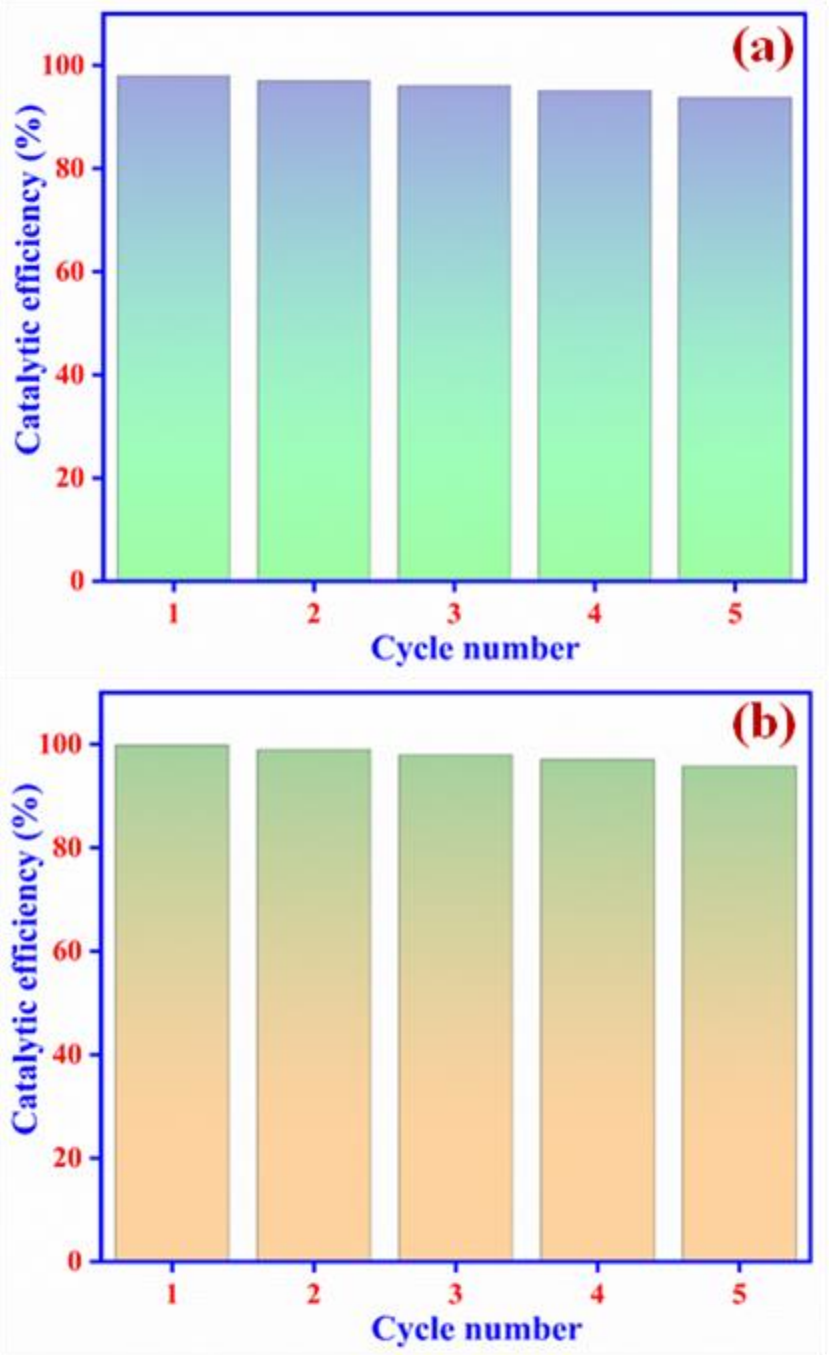

Figure S5. Recycling of In_2O_3-MF for 4-NP reduction (a) and MB degradation (b).

Table S1. The BET surface areas, pore volumes, and average pore sizes of all samples.

Sample	BET surface area (m^2g^{-1})	Pore Volume V_p (cm^3 g^{-1})	Average Pore Size D (nm)
In_2O_3-MF	486.95	0.23	0.718
In_2O_3-MR	283.26	0.13	0.767
In_2O_3-MD	182.27	0.08	0.783

Table S2. Comparison of present study with previously reported studies.

Pollutant	Photocatalyst (g/L)	Light source	Reaction Time (min)	Degradation %	Ref.
		**4-Nitrophenol**			
4-NP	Graphene/ZnO(1.0 g/L)	UV light	180	95	J. Porous Mater. 22 (2015) 797–806
4-NP	$rGO/ZrO_2/Ag_3PO_4$ (0.25 g/L)	UV light (4× 6W Hg lamp)	90	98.3	J. Hazard. Mater. 358 (2018) 416–426417
4-NP	ZnO-NCP (0.25 g/L)	UV light (75 W Hg lamp, 253 nm)	180	60	J. of Ind. Eng. Che. 20 (2014) 937–946938
4-NP	Ln-doped ZnO (1.0 g/L)	UV light (30 W Hg lamp)	195	81.63	J. Mole. Cat. A: Chem. 365 (2012) 120–127
**4-NP**	**In_2O_3-MF (0.3 g/L)**	**Xe lamp (300 W)**	**20**	**99.32**	**Present work**
		**Methylene Blue**			
MB	ZnO-GO/CGH (1.0 g/L)	Xe lamp (300 W)	180	99	RSC Adv. 9 (2019) 41209
MB	N-ZnO/GO	UV light (200 W)	35	95	Chemosphere 236 (2019), 124368
MB	RGO-Ag/ZnO (0.5 g/L)	Metal halide lamp	120	99	J. Environ. Che. Eng. 8 (2020) 1041063
MB	TiO2/ZnO/rGO (0.5 g/L)	Xe lamp (300 W)	120	99	Sep. Purif. Tech. 232 (2020), 115962.
**MB**	**In_2O_3-MF (0.2 g/L)**	**Xe lamp (300 W)**	**3**	**99.2**	**Present work**
